# Quality of care: how good is good enough?

**DOI:** 10.1186/2045-4015-1-4

**Published:** 2012-01-30

**Authors:** Mark R Chassin

**Affiliations:** 1 The Joint Commission, 1 Renaissance Boulevard, Oakbrook Terrace, IL 60181, USA

**Keywords:** Quality of care, quality improvement, improvement methods

## Abstract

Israel has made impressive progress in improving performance on key measures of the quality of health care in the community in recent years. These achievements are all the more notable given Israel's modest overall spending on health care and because they have accrued to virtually the entire population of the country.

Health care systems in most developed nations around the world find themselves in a similar position today with respect to health care quality. Despite significantly increased improvement efforts over the past decade, routine safety processes, such as hand hygiene and medication administration, fail routinely at rates of 30% to 50%. People with chronic diseases experience preventable episodes of acute illness that require hospitalization due to medication mix-ups and other failures of outpatient management. Patients continue to be harmed by preventable adverse events, such as surgery on the wrong part of the body and fires in operating theaters. Health care around the world is not nearly as safe as other industries, such as commercial aviation, that have mastered highly effective ways to manage serious hazards.

Health care organizations will have to undertake three interrelated changes to get substantially closer to the superlative safety records of other industries: leadership commitment to zero major quality failures, widespread implementation of highly effective process improvement methods, and the adoption of all facets of a culture of safety. Each of these changes represents a major challenge to the way today's health care organizations plan and carry out their daily work. The Israeli health system is in an enviable position to implement these changes. Universal health insurance coverage, the enrolment of the entire population in a small number of health plans, and the widespread use of electronic health records provide advantages available to few other countries.

Achieving and sustaining levels of safety comparable to, say, commercial aviation will be a long journey for health care--one we should begin promptly.

This is a commentary on http://www.ijhpr.org/content/1/1/3/

## Commentary

In this volume of the Journal, Jaffe and colleagues report data on the quality of community health care in Israel that demonstrate both improvement and comparability with similar data from the United States and Europe. Of the 28 measures reported, performance in 2009 was 70% or greater on fifteen measures, and 80% or greater on nine [1]. The average relative rate of improvement from 2007 to 2009 on all 28 measures was 13%. How good should we feel about these findings?

On the one hand, improvement in some areas is remarkable and so is the achievement of comparability with other health care systems on important measures of quality. These accomplishments are all the more impressive given Israel's modest overall per capita spending on health care, and because they apply, with very few exceptions, to the entire population of the country.

But can we be satisfied when the failure rate on nearly half (13) of the measures in 2009 was greater than 30%? Shouldn't we expect that all patients who could benefit from a particular health service will receive it? In general, the findings of Jaffe and colleagues mirror the state of health care quality in ambulatory and hospital care around the world--some progress in some areas over the past decade, the creation of some pockets of excellence, but many more areas of mediocrity.

Data on hospital quality are not available in Israel, but data from the United States and The Joint Commission's experience in 48 countries around the world show broadly similar patterns of quality of care. Speaking bluntly, we observe routine safety processes, such as hand hygiene, medication administration, and communication in care transitions, failing routinely. We continue to experience unacceptably high rates of entirely preventable adverse events: surgical procedures performed on the wrong patient or wrong body part, fires injuring patients in our operating theaters, and suicides of patients while they are under the care of hospitals. Patients with chronic illnesses such as heart failure, diabetes, or chronic lung disease too often experience preventable hospitalizations when errors occur in the management of their typically complex medication regimens or when their outpatient care is not sufficiently comprehensive. For example, many patients with heart failure do not understand their disease well enough to assess early warning signs of decompensation, such as weight gain and swelling around the ankles. Many do not have the specific knowledge they need to reduce the amount of sodium in their diets.

Why? What explains these quality and safety failures? Why isn't health care as safe as commercial air travel? This question is especially apropos today, because of what may fairly be described as the global awakening to the magnitude and ubiquity of health care quality problems that began about 12 years ago, initiated by a report from the US Institute of Medicine [2]. Before 2000, some could have pleaded ignorance, some were in denial, and some were inclined to see quality problems only in care provided by other physicians, hospitals, or nations. Today, few if any leaders in health care would disagree that quality problems of great magnitude are all around us, and no organization has solved more than a few of them. Internationally, it doesn't seem to matter how a nation organizes, finances, delivers, or pays for health care. The quality problems that occur during the course of health care delivery are extraordinarily similar.

I believe that health care can make the next great strides in improving quality and safety by learning from those organizations and industries that do manage serious hazards extremely well [3]. Known as "high reliability organizations," they comprise a wide variety of different human activities, including commercial aviation, nuclear power, wildland firefighting crews, and aircraft carrier flight deck operations. While few of their specific techniques can be directly applied, without significant translation, in health care settings, the fundamental principles and methods that underlie their success are highly relevant.

Broadly, the lessons from high reliability organizations suggest that health care must embrace three interrelated changes: leadership commitment to zero major quality failures, widespread implementation of highly effective process improvement methods, and the adoption of all facets of a culture of safety [4]. For many, if not most, health care organizations, these changes require major alterations in strategic planning and daily work.

Leadership commitment means that all elements of leadership (governing body, management, physician and nurse leaders) agree on a long-term goal of achieving zero major quality failures. Such a goal will not be reached quickly. But meaningful progress will be impossible without agreement on that goal. Setting targets for achieving intermediate milestones on the road to the long-term goal may be a useful strategy.

Some may object that zero is unattainable. Several years ago, it seemed impossible that intensive care units could eliminate central line bloodstream infections or ventilator-associated pneumonia. Yet that is exactly what many have achieved and maintained. I do not underestimate the degree of difficulty many organizations will have with this task. Some may need to educate their governing bodies about the magnitude of their quality problems. Some chief executive officers may not be up to the job. Some organizations may find a dearth of physicians or nurses ready to lead in this direction. Building this leadership consensus will tax the change management capacities of many health care organizations. But it is an essential first step.

If we aim for major, unprecedented improvements in quality, we must understand how our current methods of improvement fall short. For many years we have tended to rely on a remarkably singular technique to improve health care quality: the "best practice." This approach works something like this. If one organization reports success in dealing with a recalcitrant problem (say, hand hygiene compliance) with a specific 5-step method, we call that 5-step method a "best practice" and expect all other organizations to deploy it the same way as the original developer and to benefit to exactly the same degree. While this approach has produced some improvement, its limitations have blocked our progress in getting to zero. Complex problems require more sophisticated problem-solving methods than "best practices" permit.

Approaches like lean, six sigma, and systematic approaches to change management are far better suited to the complexities of problems such as communication failures in care transitions and wrong site surgery. In brief, using these tools enables organizations to measure the magnitude of the problem reliably and to identify the key root causes that explain the majority of failures. Improving hand hygiene compliance may require placing hand gel dispensers in convenient locations or improving the education of specific kinds of caregivers about when hand cleaning is necessary. It may also require creating an environment or culture in which all caregivers are encouraged to point out lapses in compliance by anyone else and are protected when they do so. Each of these root causes requires a very different intervention to eradicate. Typically, three or four specific key root causes explain a majority of the failures. However, experience has shown that different organizations (e.g., hospitals) usually exhibit very different groups of key root causes [5]. This complexity goes a long way toward explaining why the "best practice" approach to improvement so often produces mediocre results. Each organization must identify what its key root causes are for a particular problem so it can customize a set of interventions targeted specifically to those root causes. This approach is highly effective in addressing only the most important causes. It is also highly efficient, because it avoids the wasted effort that attends to deploying parts of a "best practice" that are aimed at causes that are not present.

The last component of the triad of changes is arguably the most difficult for health care organizations to achieve: a fully developed culture of safety [6]. High reliability organizations sustain their high levels of excellence in large part because all employees are obligated and encouraged to identify and report the smallest safety problems, for example, faulty equipment maintenance, safety protocols that are not properly implemented, or unsafe conditions, as soon as they are apparent. Management uses highly effective tools to fix the problems long before they are likely to lead to harm, when they are far easier to fix, and reports the improvements back to the frontline workers who reported them. Such improvements reinforce the trust of employees in the organization and lead to the continuous identification and remediation of problems that are far upstream from harm. Contrast this scenario with health care, where we are most often in the position of looking backward after a patient has been harmed by a preventable adverse event, trying to figure out why it happened, and devising action plans to prevent future occurrences.

The challenges involved in creating a fully effective safety culture for health care are daunting. They include establishing mechanisms that, on the one hand, encourage and do not punish reporting of unsafe conditions and behaviors, and, on the other hand, hold individuals accountable for adherence to safety protocols and procedures. Balancing learning from the reporting of blameless acts with accountability for blameworthy ones is crucial. Health care organizations must eliminate the widespread occurrence of intimidating behaviors that block reporting [7]. They must also adopt transparent methods for distinguishing the blameless from the blameworthy and apply them equitably across all groups of caregivers.

What is the relevance of this model of improvement for Israel? I believe it is highly relevant. Unlike the United States but in common with European health systems, Israel has virtually eliminated financial barriers to health care at the individual patient level. Remaining barriers in Israel will have more to do with systems of care and how well they function than how they are financed. Further, since virtually all Israelis are members of health plans and because the use of electronic health records is so widespread, Israel has the infrastructure to support a systemwide commitment to high reliability healthcare. This opportunity stands in stark contrast, for example, to the United States with its extreme fragmentation of health care, where several independent physicians may be caring for the same patient and not communicate with or even be known to each other. This endemic lack of capacity to coordinate care has led many observers to refer to US health care as a "nonsystem."

I suggest that one significant next step for Israeli health plans might be to examine some of the lower-performing measures, perhaps influenza vaccination for individuals with asthma (40% in 2009) or adequate blood pressure control in diabetics (68.6% in 2009) [1]. Choosing which measures in which to invest additional improvement effort will require an important exercise in priority setting. A number of important factors should be considered. How confident are we in the quality of the measure? Said another way, how sure are we that improving performance on the measure will lead directly to improved health outcomes for patients? How much will it likely cost to achieve those improved outcomes? Are the benefits commensurate with the costs?

Using the tools noted above, improvement projects would identify exactly why these processes do not exhibit much higher levels of performance. These causes would be expected to differ by site of care, by patient socioeconomic status, and by race or ethnicity. This approach to improvement is particularly useful in community-based care, where patient choices and behaviors heavily influence adherence to effective treatments. While providers cannot control behaviors that lead to lack of adherence, they can learn why they occur and take steps to remedy underlying problems. For example, the heart failure patient who experiences frequent exacerbations because she is ingesting too much salt in her diet may not know that that behavior leads directly to the fluid overload that worsens her condition. She may not know exactly which foods she should avoid to reduce her sodium intake or how to substitute acceptable low-sodium foods for her present dietary choices. It is this kind of knowledge that is essential to designing effective improvement strategies.

Interventions targeted to the most important specific causes at each site of care would be developed, tested, and proved to reduce the failure rates. The knowledge would be disseminated, not as a list of "best practices" to be implemented everywhere, but rather as solutions targeted to specific causes of failure. Effective dissemination requires each site of care to first identify causes of failure among its patients and then to implement the set of interventions already proven to remediate those specific causes. Perhaps each of Israel's four health plans could take on one such initiative to improve on one low-performing measure, with the methods overseen by a neutral third party to assure comparability. The end result would be tools that all plans (and other organizations) could use to improve substantially on four key quality measures.

Health care quality and safety failures harm patients every day in every developed nation around the world. Surely, the current state is not good enough. Responsibility for dramatic improvement rests with all organizations that deliver health care and with those that support and regulate them. The three-part strategy of leadership commitment to zero major quality failures, broad employment of sophisticated problem solving tools (lean, six sigma, and change management), and the creation of all the elements of a safety culture is a model that offers great promise. The journey to high reliability may be long and difficult for health care. There is no better reason to begin it today.

## Competing interests

The author declares that they have no competing interests.

## Author's information

Mark R Chassin is the president of The Joint Commission and also president of the Joint Commission Center for Transforming Healthcare.
